# Agreement and utility between registry and trial mortality data - a data utility comparison in the BOSS trial

**DOI:** 10.1186/s13063-026-09605-7

**Published:** 2026-03-17

**Authors:** Alex Zimmermann, Oliver Old, Hugh Barr, Sharon B. Love, M. Sofia Massa

**Affiliations:** 1 Oxford Clinical Trials Research Unit, Centre for Statistics in Medicine, Department of Orthopaedics, Rheumatology, and Musculoskeletal Sciences, University of Oxford, Oxford, OX3 7LD UK; 2 Gloucestershire Hospitals NHS Foundation Trust, Gloucester, UK; 3 MRC Clinical Trials Unit at UCL, Institute of Clinical Trials and Methodology, 90 High Holborn, London, WC1V 6LJ UK

**Keywords:** Healthcare Systems Data, Data Utility Comparison Studies, Barrett’s oesophagus, Overall survival, Mortality data, Randomised Controlled Ttrials

## Abstract

**Background:**

Healthcare Systems Data (HSD) are increasingly used in RCTs to supplement data required for clinical trial outcomes; however, the true quality and utility of this data remain unclear. In clinical trials when data discrepancies are present, rules of data integration must be in place to handle the differences; however, there is little evidence as to the best approach for how these principles are set. The purpose of this study is to conduct a comprehensive data utility comparison of HSD mortality data and trial-specific mortality data collected in the BOSS trial.

**Methods:**

Trial-specific and HSD mortality data collected in the BOSS trial were compared to assess levels of agreement between death status, death dates, and causes of death. Potential sources of data inconsistencies were examined to determine underlying patterns between HSD and trial-specific data discrepancies. HSD and trial-specific data were combined through five data integration approaches to assess their impact on the primary outcome of overall survival.

**Results:**

Death status and death dates were similar between trial-specific and HSD data (Cohen’s Kappa statistic 0.866, 95% CI 0.842 to 0.889); however, more than 100 participants were recorded with inconsistent death statuses, and the median difference between different death dates was almost a year (340 days, IQR: 11 to 865 days). All data integration approaches contributed to similar treatment effect estimates, and none changed the results of the trial. The treatment effect estimations from either source exclusively were comparable (HSD Hazard Ratio 1.01, 95% CI 0.85 to 1.20; trial-specific Hazard Ratio 0.89, 95% CI 0.75 to 1.06), and when both sources were integrated to produce hazard ratio estimates, the results were almost identical regardless of the approach taken.

**Conclusions:**

In a direct comparison of mortality data between HSD and trial-specific data for the BOSS trial, both sources were mostly accurate and complete. Trial results were not impacted by different approaches of data integration and were generally strengthened by combining all data sources. This should be always encouraged when trial data and HSD are both collected.

**Trial registration:**

ISRCTN54190466. Registered on 8 January 2008.

## Introduction

A vast landscape of UK data collection structures has developed in recent decades leading to the capacity for collecting considerable quantities of data with ease. The rise of ever-growing datasets from across therapeutic areas has produced a new and potentially viable source of healthcare data. Recent developments in Healthcare Systems Data (HSD) have led to discussion around the potential addition of HSD to trial-specific data, specifically in the context of randomised controlled trials (RCTs) [1, 2]. This data integration aims to form a stronger evidence base for clinical trials by providing more comprehensive datasets from which to draw results [3]. Further, HSD, such as electronic health records and registries, have the potential to supplement clinical trials by improving the quality of data used, reducing costs of data collection, providing an expanded pool of clinical outcomes available, and increasing convenience to trial teams [4–6]. As a result, HSD is increasingly used by clinical trials in the UK, with around two thirds (62%) of NIHR-funded RCTs commencing after 2019 planning to utilise RCHD as outcome data [7]. This has increased from before 2019 when 47% of NIHR-funded RCTs used RCHD outcome data, and only 3% for all UK-based RCTs between 2013 and 2018 [1, 8]. In the context of RCTs, HSD provides additional utility and understanding to a trial’s results; however, to be deemed high-quality, it is essential that the data is accurate, complete, and verifiable.

Despite the increased use of HSD in RCTs, there is still no conclusive agreement over the quality of HSD. Mortality-related outcomes make up 76% of all trial outcomes collected from HSD. It is therefore essential that especially death data (e.g. death status, date of death, and cause of death) is of high quality such that the health research utilising these potentially rich data sources are not undermined by the quality of their data [1]. Despite the shared underlying principles of HSD and trial-specific data collection, the presence of data discrepancies is inevitable, and this has led to uncertainty in clinical trials where they are observed [9]. In such trials, decision principles must be in place to handle the differences; however, there is little evidence or guidance as to the best approach for how these principles are set: what do we do when a participant dies in one data source but not the other? What do we do when the recorded dates of death are different across sources?

### The BOSS trial

The Barrett’s Oesophagus Surveillance versus Endoscopy at Need Study (BOSS) was a phase III, multicentre Randomised Controlled Trial which randomised 3452 UK-based, adult, Barrett’s oesophagus patients from 132 sites between March 2009 and November 2011 [10]. Participants were randomised to two-yearly endoscopic surveillance or an endoscopy ‘at need’ only. Local trial sites identified eligible participants from new local diagnosis of Barrett’s oesophagus or from existing disease registers. All trial-specific data were collected centrally from sites using Case Report Forms (CRFs) on a regular basis throughout trial follow-up.

HSD from the Medical Research Information Service (MRIS) (now managed by NHS Digital) was collected via linkage of all participants to the NHS (using NHS number, surname, forename, sex, date of birth, last known address and last known postcode) at the point of randomisation into the BOSS trial and death data was provided by MRIS up to October 2020. Trial-specific mortality data was collected up to January 2024 in the BOSS trial. For the purpose of this study, trial-specific death data was only considered up to the end of MRIS data provision (October 2020). This was to ensure death data from both sources was censored at the same time horizon and enable a fair assessment of the comparability between the data sources. In total, 11 years of follow-up mortality data was collected from each source to primarily assess the impact of regular endoscopies on patients’ overall survival in the intention-to-treat population. In 2021, Love et al., compared the trial-specific death data in the BOSS trial with NHS Digital data up to 2018 [11]. This case concluded that more assessments of registry data used in RCTs should be performed to gain a more complete picture of HSD utility for trials. The comparison in [11] was conducted prior to the end of the trial, when data collection was incomplete and it was not possible to present the effect of trial data on the BOSS trial’s final results. Further, there has been a greater emphasis in recent years calling for research to conduct comparisons of HSD for use in clinical trials amongst the scientific community [12]. 

This case study aims to enhance the understanding of data quality from HSD and UK-based RCT sites and strengthen the evidence base for the quality of data that underpins most patient-level medical research. In order to do so, we extend upon the work by Love et al. [11] and conduct a comprehensive comparison of HSD and trial-specific mortality data in the BOSS trial using the full 11 years of follow-up for which we have both sources of mortality data. We set out to assess both sources for levels of agreement (accuracy and completeness), potential sources of data inconsistencies, and evaluate five data integration approaches to assess their impact on the primary outcome of overall survival.

## Methods

The mortality status retrieved from HSD and trial-specific data in this study’s comparison includes death status (alive/dead), death date, and cause of death. CRFs were used to collect trial-specific data, whereas HSD was obtained from MRIS which is generated from Office for National Statistics (ONS) data extracts to NHS Digital. The HSD cause of death was classified using the International Classification of Diseases (ICD)−10 [13] codes whereas the corresponding cause of death information was categorised from free text in the Case Report Forms from recruitment centres. Participants who withdrew completely from data collection in the BOSS trial were censored in both sources at the dates of withdrawal. Mortality status was compared across both sources for accuracy and completeness, potential sources of bias, and data utility as described in the following paragraphs.

### Agreement

The extent to which data sources agree and disagree was examined by cross-tabulating participant status (alive/dead) as reported by each source. An inter-rater reliability estimate was calculated using Cohen’s Kappa statistic and 95% Confidence Interval (CI). The observed survival times and rates of deaths were presented by each source to understand when deaths are reported to have occurred. For participants whose death was recorded in both datasets, a scatterplot of death dates captured in each source was produced to represent the time between deaths recorded. Further, the time gaps between deaths recorded were also plotted in order of the size of the gap. Additionally, for participants who had conflicting death dates between the sources, the timeline from the first date of death recorded (from either source) to the second date of death was plotted and categorised by which source the first death was reported by. A Cohen’s Kappa statistic was also estimated to summarise any differences in causes of death recorded.

### Potential sources of disagreement

Baseline characteristics were cross-tabulated for participants with deaths recorded by either source exclusively, deaths recorded by both sources, and all participants. Associations between baseline variables and source of death status (binary variable categorised as 0 if the source of death was HSD and 1 if the source of death was trial-specific) were also explored by examining participants with discordant death statuses exclusively. A logistic regression model was used in this subset of participants to understand if there were discernible factors associated with patterns of heterogeneous data collection between sources. The potential factors chosen were the baseline characteristics considered most clinically relevant to Barrett’s oesophagus: age at randomisation (years), age at Barrett’s diagnosis (< 65 years, ≥ 65 years), sex (male/female), body mass index (kg/m^2^), Barrett’s newly diagnosed (< 4 months/≥ 4 months), and Barrett’s segment length (< 2 cm, ≥ 2 cm and ≤ 3 cm, > 3 cm and ≤ 8 cm, > 8 cm). Three of these variables were also randomisation factors (age at Barrett’s diagnosis, Barrett’s segment length and Barrett’s newly diagnosed).

### Data utility

The extent to which additional utility was gained through the addition of HSD to trial-specific data was assessed using the trial’s primary outcome: overall survival (defined as time from randomisation to death from any cause, see also [10]). Five approaches to the integration of the two data sources aiming to represent the equivalent true event were considered and time to death was derived using: (1) HSD mortality data only; (2) trial-specific mortality data only; (3) the combination of mortality data sources prioritising HSD death dates when both dates were present (as applied in the analysis of the BOSS trial [10]); (4) the combination of mortality data sources prioritising trial-specific death dates when both dates were present; and (5) combining data sources averaging time to death when both sources were present. To assess the implications of the approaches on trial results, hazard ratios of the treatment interventions were estimated using a Cox proportional hazards model adjusted for the same variables as in the primary outcome of the BOSS trial: age at Barrett’s diagnosis (< 65 years, ≥ 65 years), sex (male/female), body mass index (kg/m^2^), Barrett’s diagnosis date (< 4 months/≥ 4 months), Barrett’s segment length (< 2 cm, ≥ 2 cm and ≤ 3 cm, > 3 cm and ≤ 8 cm, > 8 cm), low grade dysplasia (yes/no), and indefinite dysplasia (Yes/No). Hazard ratios and 95% CIs estimated for all five data integration approaches were presented in a forest plot.

All analyses were performed using R 4.3.1 and RStudio 2023.09.0.

## Results

### Agreement

The main results of the BOSS trial, including the CONSORT diagram, baseline characteristics by treatment arm, and outcome results are reported elsewhere [10].

Most participants remained alive throughout the course of data follow-up (2859/3542, 83%), and both data sources recorded comparable numbers of deaths: HSD captured 531 (15%), and trial-specific data captured 534 (15%) deaths, and they agreed on 472 (14%) deaths (Table 1). Whilst the majority of death statuses matched between sources, a notable total of 121/593 (20%) participants were reported to have a death by one source but not the other (Inter-rater reliability Cohen’s Kappa statistic: 0.866, 95% CI 0.842 to 0.889). There were 156/3542 (5%) participants who withdrew completely from data collection and were censored in both sources at their dates of withdrawal.

**Table 1:** Death status reported by data source

		**Healthcare Systems Data**		
		**Alive**	**Dead**	**Total**
**Trial-specific **	**Alive**	2859 (83%)	59 (2%)	2918 (85%)
	**Dead**	62 (2%)	472 (14%)	534 (15%)
	**Total**	2921 (85%)	531 (15%)	3452 (100%)

Cohen’s Kappa statistic: *κ* = 0.866, 95% CI 0.842 to 0.889

The agreement on cause of death was very poor (κ: 0.05, 95% CI 0.03 to 0.08); however, this was due to the poor cause of death captured in the trial-specific data, as only 16% of deaths had an identified cause of death (compared to 97% in HSD) (Table 2).

**Table 2:** Causes of death as reported by data source

Cause of death		Healthcare Systems Data cause of death							
		Oesophageal cancer	Gastric cancer	Other cancers	Disease of the circulatory system	Other	Not recorded	No death	Total
Trial-specific cause of death	Oesophageal cancer	20	0	0	0	2	0	1	23 (3.9%)
	Gastric cancer	0	0	0	0	0	0	0	0 (0.0%)
	Other cancers	0	0	0	0	0	0	9	9 (1.5%)
	Disease of the circulatory system	0	0	0	0	0	0	11	11 (1.9%)
	Other	0	0	0	0	0	0	20	20 (3.4%)
	Not recorded	12	1	137	117	168	15	21	471 (79.4%)
	No death	1	0	13	9	16	20	0	59 (9.9%)
	Total	33 (5.6%)	1 (0.2%)	150 (25.3%)	126 (21.2%)	186 (31.4%)	35 (5.9%)	62 (10.5%)	593 (100%)

Cohen’s Kappa statistic: 0.052, 95% CI 0.029 to 0.075

**Fig. 1:**
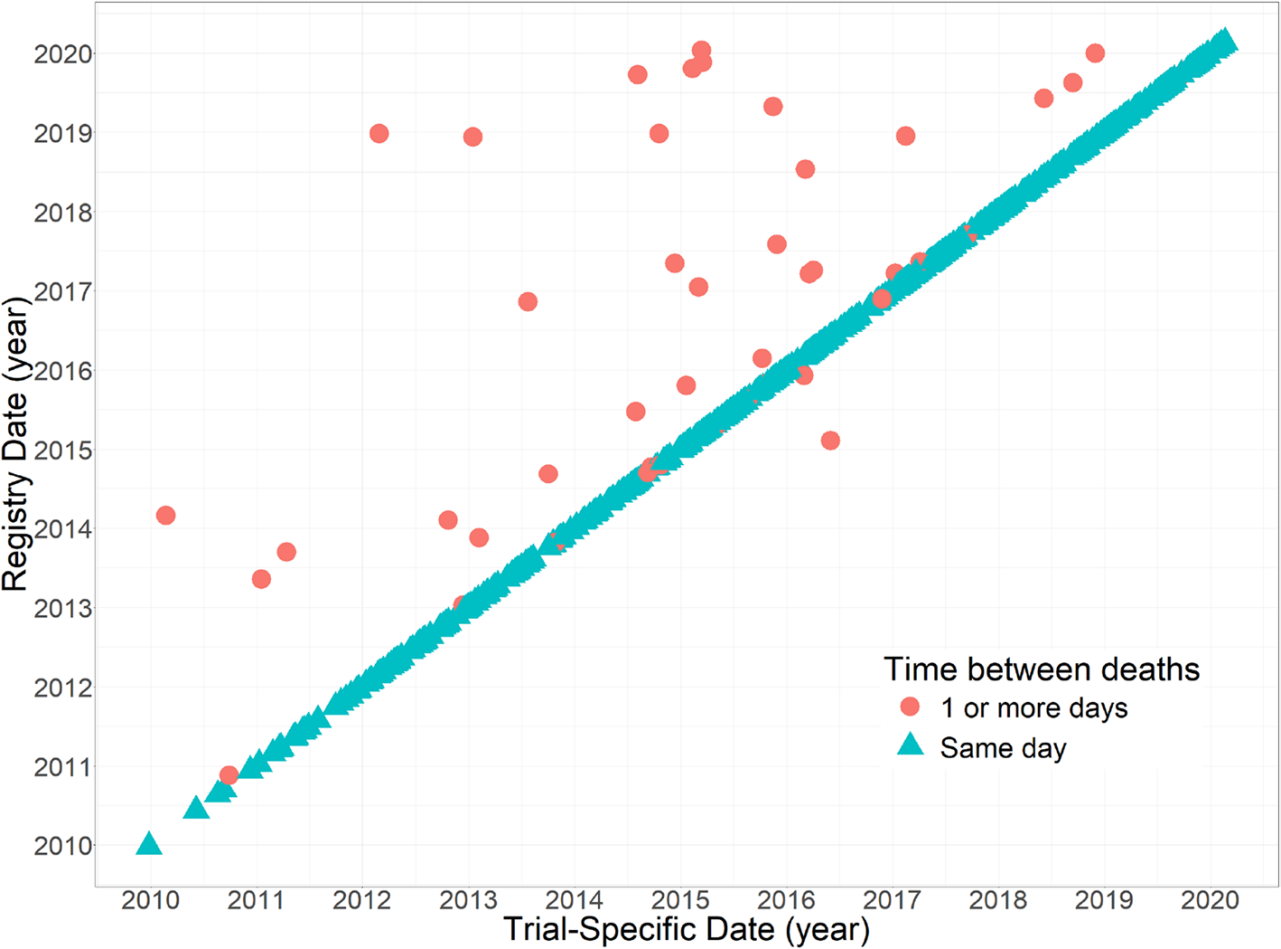
Scatterplot of Healthcare Systems Data and trial-specific participant death dates data for all individuals with a recorded death in both data sources. The discrepancies between death dates represent the time between two death dates for the same participant

**Fig. 2:**
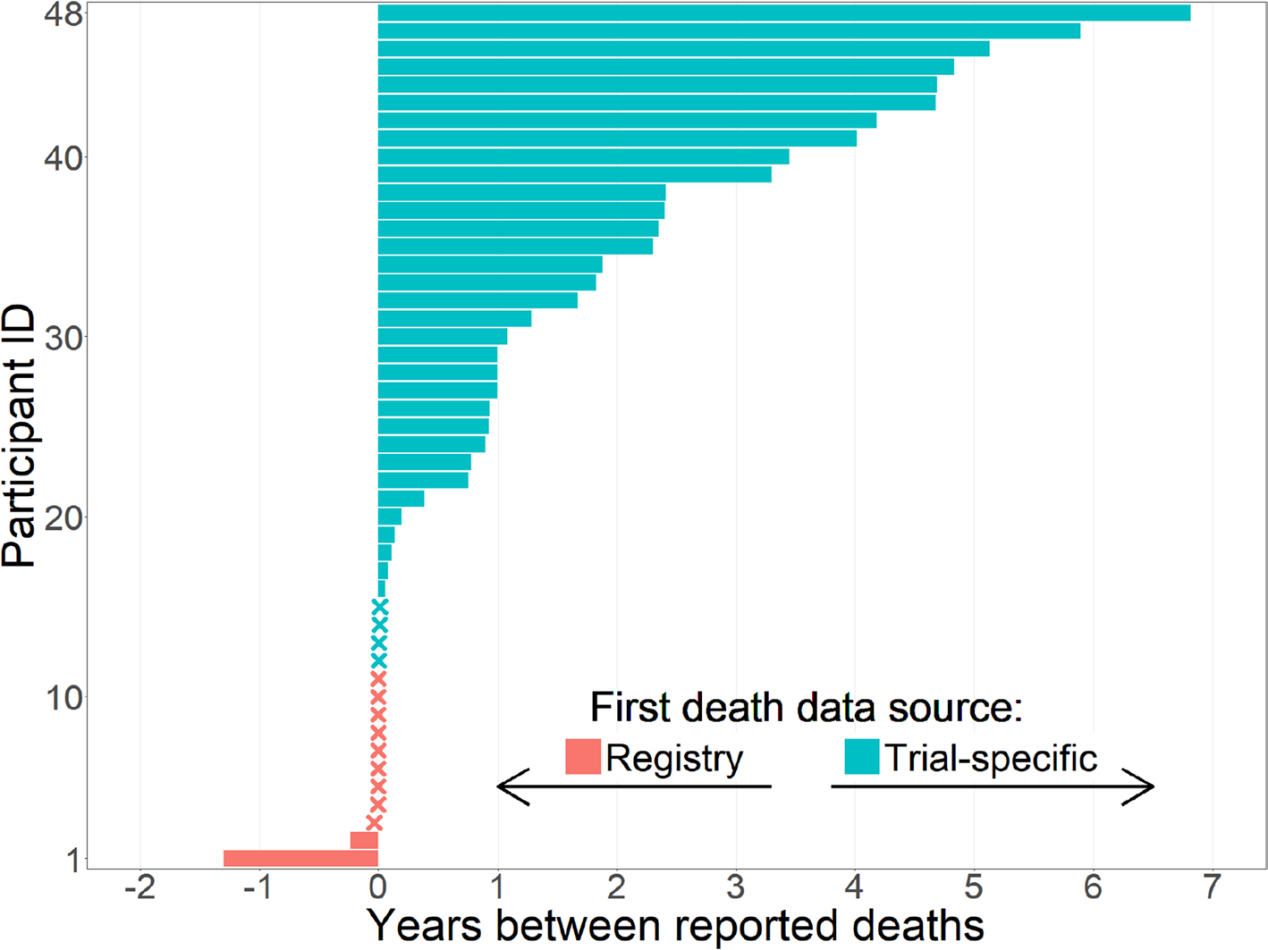
Time between participant death dates according to HSD and trial-specific data

Follow-up times were similar between sources (median 9.6 years) and made no difference to the rate of deaths estimated (Table 3). In the majority of cases when a death was captured in both sources, the date of death recorded was the same date (424/472, 90%). Despite strong correlation between death dates (*r* = 0.95, 95% CI 0.94 to 0.96), when discrepancies between dates were present the dates were often widely distinct from each other (Fig. 1). Whilst the minimum time between death dates for the same participant was 1 day, the maximum was as large as 6.8 years, and the median difference between dates was 340 days (interquartile range: 11, 865 days) (Fig. 2). The trial-specific date was before the HSD in most cases of date discrepancies (37/48, 78%). In total, 169/3452 (5%) of all participants and 169/593 (29%) of all potential deaths were either discrepant due to death status or death date.

**Table 3:** Survival times by data source

	**Healthcare Systems Data**	**Trial-specific**
**Follow-up time (years)**		
**All participants**		
*N*	3452 (100%)	3452 (100%)
Median (SD)	9.57 (2.16)	9.58 (2.18)
(Q1, Q3)	(9.06,10.15)	(9.08,10.15)
[Min, Max]	[0.01,11.59]	[0.00,11.59]
**Participants who died**		
*N*	531 (15%)	534 (15%)
Median (SD)	5.80 (2.47)	5.79 (2.67)
(Q1, Q3)	(3.78,7.46)	(3.60,7.79)
[Min, Max]	[0.01,10.28]	[0.00,10.53]
** Rate of deaths** ^**1**^	1.715	1.715

^1^Deaths per 100 patient-years

### Potential sources of disagreement

Most baseline characteristics were well balanced between data sources used, showing no evidence of an association between a characteristic and the likelihood of a death being reported in one source but not the other (Table 4). Age at randomisation was the only characteristic where the odds of a reported death in HSD or trial-specific data were heterogeneous, with older participants’ deaths having a higher chance of being reported in HSD than by trial-specific data (Odds Ratio: 0.92, 95% CI 0.85 to 0.98; *p* = 0.018). The odds ratios reported in Table 4 should be interpreted with some caution due to the limited number of discordant events (*n* = 121).

**Table 4:** Baseline characteristics by death status source. Summary statistics are *n* (%) for categorical data and mean (standard deviation), Q1 (25% percentile) and Q3 (75%percentile), minimum and maximum value for continuous data

**Baseline characteristic**	All trial participants *N* = 3452	HSD and trial-specific death *N* = 472	HSD death only *N* = 59	Trial-specific death only *N* = 62
**Age at diagnosis (years)** ^**1**^				
< 65 years	2325 (67%)	198 (42%)	22 (37%)	34 (55%)
≥ 65 years	1127 (33%)	274 (58%)	37 (63%)	28 (45%)
			Odds Ratio (< 65 years vs. ≥ 65 years): 1.55, 95% CI 0.48 to 5.21	
**Length of Barrett’s segment** ^**1**^				
< 2 cm	123 (4%)	11 (2%)	0 (0%)	2 (3%)
2–3 cm	1407 (41%)	153 (32%)	22 (37%)	22 (35%)
> 3–8 cm	1574 (46%)	231 (49%)	27 (46%)	32 (52%)
> 8 cm	348 (10%)	77 (16%)	10 (17%)	6 (10%)
			Odds Ratio (2–3 cm vs. > 3–8 cm): 1.64, 95% CI 0.69 to 3.95	
			Odds Ratio (2–3 cm vs. > 8 cm): 0.92, 95% CI 0.25 to 3.22	
**Barretts newly diagnosed (< 4 months prior)** ^**1**^				
Yes	760 (22%)	107 (23%)	14 (24%)	17 (27%)
No	2692 (78%)	365 (77%)	45 (76%)	45 (73%)
			Odds Ratio (< 4 months vs. > 4 months): 0.64, 95% CI 0.24 to 1.67	
**Sex**				
Male	2462 (71%)	361 (76%)	37 (63%)	44 (71%)
Female	990 (29%)	111 (24%)	22 (37%)	18 (29%)
			Odds Ratio (male vs. female): 0.72, 95% CI 0.29 to 1.76	
**Age at randomisation (years)**				
Mean (SD)	63.2 (10.5)	70.5 (9.3)	71.8 (8.3)	66.3 (8.7)
(Q1, Q3)	(56.7, 70.5)	(65.2, 77.2)	(66.3, 78.5)	(61.4, 72.9)
[Min, Max]	[19.2, 92.0]	[29.4, 90.9]	[53.9, 89.2]	[44.6, 83.6]
			Odds Ratio (years): 0.92, 95% CI 0.85 to 0.98	
**Body mass index (kg/m** ^**2**^ **)**				
Mean (SD)	28.4 (4.9)	27.6 (4.9)	27.7 (6.8)	28.2 (4.9)
(Q1, Q3)	(25.1, 30.8)	(24.2, 29.9)	(23.1, 30.0)	(25.5, 29.8)
[Min, Max]	[15.6, 60.9]	[15.6, 58.7]	[17.0, 56.6]	[18.3, 43.5]
Missing	39 (1%)	5 (1%)	1 (2%)	3 (5%)
			Odds Ratio (kg/m^2^): 1.02, 95% CI 0.95 to 1.09	

^1^Stratification factors

Odds ratios adjusted for all other characteristics shown in table

Length of Barrett's segment (<2cm) odds ratio failed to converge to a stable estimate due to lack of events in this group

### Data utility

**Fig. 3:**
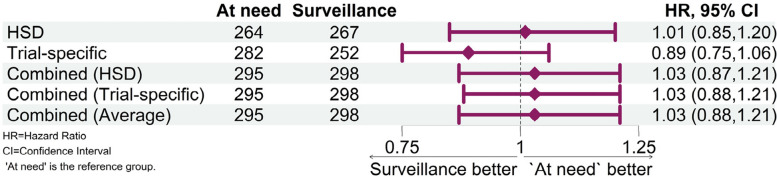
Forest plot of hazard ratio estimates between two-yearly endoscopic surveillance or an endoscopy ‘at need’ treatment arms by the five data integration approaches. Overall survival was derived using: HSD: HSD mortality data only ; Trial-specific: trial-specific mortality data only ; Combined (HSD): the combination of mortality data sources prioritising HSD death dates when both dates are present; Combined (Trial-specific): the combination of mortality data sources prioritising trial-specific death dates when both dates were present; Combined (Average): when both HSD and trial-specific deaths were collected, the mean time to death from both sources was used for the analysis

Out of the five data integration approaches applied to the mortality data, none changed the results of the trial (Fig. 3). All the estimated overall survival hazard ratios between the endoscopy at need and two-yearly endoscopic surveillance arms were similar and close to null value. The exclusive use of trial-specific data produced the lowest hazard ratio estimation; however, it would not have led to a different clinical conclusion in this scenario. It is interesting to note that in all three approaches utilised for combining sources, hazard ratio estimates, and confidence intervals were conducive to almost identical results.

## Discussion

This comprehensive comparison of mortality data within the BOSS trial has highlighted a strong correlation between HSD and data collected from sites, but also potential discrepancies that can arise between them. The inter-rater reliability of participants’ statuses at the end of follow-up was strong, and the dates of deaths recorded were very highly correlated, but discrepancies were also present.

With over 20% of participants who were recorded as dead in either source only being recorded in one, this could show a lack of accuracy and completeness in both sources. Whilst the large gaps between deaths being captured is problematic, most dates were identical, and many of those which did not match were only very few days apart and more acceptable due to the potential logistical challenges involved in data collection.

Despite clear differences between HSD and data collected from sites, this comparison is limited by the age of BOSS. BOSS is the largest randomised controlled trial undertaken in Barrett’s oesophagus, with more than 39,500 patient-years of follow-up; however, the trial initiated follow-up in 2009. Since the start of the trial, data collection processes in RCTs have been able to develop, and it is possible that future trials will process data collected from sites in a more structured manner such as using electronic data collection systems. The trial team recognised this concern and requested sites to record mortality statuses on all participants according to the most up-to-date knowledge at the end of the trial such that the trial-specific data was as accurate and complete as possible, mitigating data collected earlier in the trial via a potentially older process.

There are a variety of potential causes of data entry errors which could lead to discrepancies in HSD and trial-specific data; however, it is important to examine whether the cause of these inaccuracies is random human error or whether a more systematic pattern is observable. Age was the only factor associated with different mortality statuses across sources. In order to assess whether this result is spurious, there should be some heuristic reasoning to support a potential conclusion that age affects the chances of underreporting trial-specific data/overreporting HSD. It is possible that RCT sites struggle to follow up and capture mortality statuses of elderly patients, whereas a registry could capture this data more easily; however, further research is needed in this area.

The more we understand about the quality of HSD used in RCTs and the approaches used in their integration, the more opportunities will be given to RCTs. A key concern in an RCT enhanced with HSD is the direct impact on outcomes when there is some degree of non-uniformity between sources. It is clear that within the context of this RCT, there was no evidence of a difference in overall survival between treatment arms using any approach for data integration. It is plausible in another setting for the exclusive utilisation of one source to contribute to results which could potentially lead to different conclusions depending on the source used; however, the integration of data creates the most complete picture of mortality within a trial’s lifetime. The methodologies applied to combining sources appeared to have negligible impact on the results observed in this scenario. It is therefore clear that wherever HSD is accessible, combining sources is favourable unless one source has been declared of higher quality than the other. Deciding the rules of priority is more difficult when more discrepancies are present, and these priorities should be pre-specified, and choices discussed before the beginning of data collection.

HSD is increasingly used in RCTs, and relevant guidance is being developed to ensure the effective and transparent use of HSD in RCTs [7, 14, 15]. Further, there have been encouragements to compare the HSD collected in trials with the corresponding trial-specific data, and examples of some trials where estimates of treatment effects have been compared using HSD and trial-specific data sources [2, 12]. This comprehensive comparison contributes to the existing understanding of the extent of agreement and utility gained through use of these data sources by demonstrating the comparability, limitations, and impact they may have on treatment effect estimates.

## Conclusions

In a direct comparison of data between HSD and trial-specific data, the sources were mostly accurate and complete, and there was little evidence of clinical factors causing bias between them. Despite strong agreement between sources, there remained some considerable differences in death statuses and dates. The addition of mortality data from a HSD source to trial-specific data in the context of an RCT provided a series of overall survival treatment effect estimates comparable to trial-specific sources alone. Trial results were strengthened by combining sources, and this should be encouraged where possible; however, it was not relevant which source was given priority in the presence of discrepancies because varying approaches led to almost identical results. HSD should be considered a helpful addition to the evidence base of future RCTs; however, minor discrepancies should be expected, and it is strongly suggested that pre-specified plans for managing such differences should be employed.

## Data Availability

The BOSS trial began in 2009 before International Committee of Medical Journal Editors requirements to have a data sharing agreement. No data sharing agreement was in place when enrolling patients into the trial. All data requests should be submitted to the corresponding author for consideration. Access to anonymised data may be granted after review and having a data sharing agreement in place. Individual participant data will not be shared.

## Abbreviations

HSD: Healthcare Systems Data
RCT: Randomised Controlled Trials
CI: Confidence Interval
IQR: Interquartile Range
UK: United Kingdom
MRIS: Medical Research Information Service
NHS: National Health Service
ICD-10: International Classification of Diseases
BOSS: Barrett’s Oesophagus Surveillance versus Endoscopy at Need Study
